# 3D Printing in Heterogeneous Catalysis—The State of the Art

**DOI:** 10.3390/ma13204534

**Published:** 2020-10-13

**Authors:** Elżbieta Bogdan, Piotr Michorczyk

**Affiliations:** Institute of Organic Chemistry and Technology, Faculty of Chemical Engineering and Technology, Cracow University of Technology, Warszawska 24, 31-155 Kraków, Poland; piotr.michorczyk@pk.edu.pl

**Keywords:** 3D printing, additive manufacturing, monolithic catalysts

## Abstract

This paper describes the process of additive manufacturing and a selection of three-dimensional (3D) printing methods which have applications in chemical synthesis, specifically for the production of monolithic catalysts. A review was conducted on reference literature for 3D printing applications in the field of catalysis. It was proven that 3D printing is a promising production method for catalysts.

## 1. Introduction

Three-dimensional printing is a popular term for additive manufacturing (AM) [1]. There are many definitions of AM in use. The official one proposed by the Technical Committee of ASTM International in NF ISO/ASTM 52900 is worded as follows: “Additive manufacturing is a process of joining materials to make objects from 3D (three-dimensional) model data, usually layer upon layer, as opposed to subtractive manufacturing methodologies” [2]. Other derivative terms are in use. One of the important ones which was used first is rapid prototyping (RP) [1]. RP is a blanket term for a number of technologies used for manufacturing precision parts (prototypes) directly from their digital models, in a short time frame and with low human intervention [3]. The same technologies can be applied for the rapid manufacturing (RM) of finished products and rapid tooling (RT), a process of fast production of processing tooling, like injection molds [4]. Advertised as a replacement for traditional subtractive methods, 3D printing has attracted great attention from the media and the scientific community lately [5,6].

**Figure 1:**
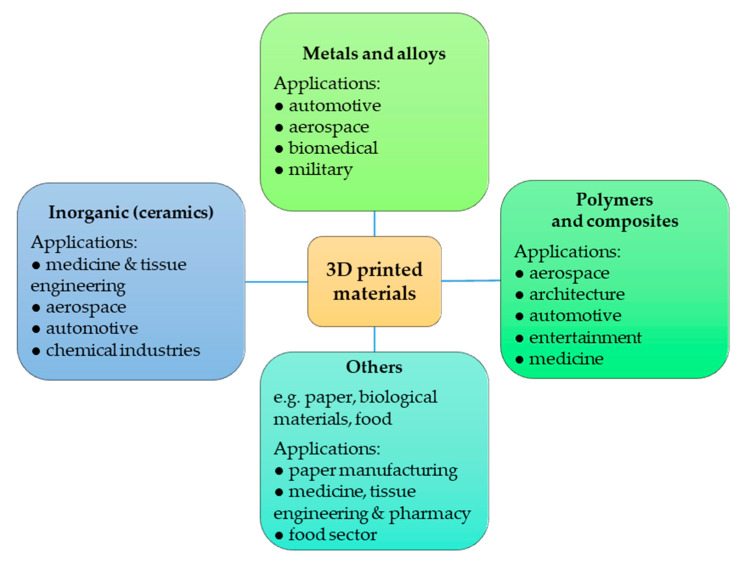
Three-dimensional-printed materials and their applications.

AM can be applied in the manufacturing of monoliths, which are systems comprising functional microchannels with a regular three-dimensional structure. They can replace conventional catalysts and chemical reactors while helping to overcome multiple problems posed by traditional systems. AM-developed products can be designed precisely in every detail and adapted to specific processes [7,8,9]. Three-dimensional printing allows the building of complex 3D structures from many different materials, as presented in Figure 1. This technology helps to produce catalysts with the desired properties. Many chemical and industrial processes exist which can be enhanced with 3D printing, which makes the latter a very promising field of research [8,10,11].

In this review, we provide general information on the stages of the 3D printing process, 3D printing methods, and applications in the field of heterogeneous catalysis. We distinguish structures manufactured directly and indirectly, including monolithic catalysts, static mixers, and reactionware. We describe structures made of various materials such as: ceramics (Al_2_O_3_, SiO_2_, TiO_2_, other oxides, SiC), zeolite (ZSM-5, Y), metal (steel, titanium alloy, cobalt-chromium alloy), carbon, and polymer (methacrylate, silicone), containing some active phases. We focus on the synthesis of 3D structures and their performance in catalytic processes.

## 2. 3D Printing Process

### 2.1. Stages of 3D Printing Process

**Figure 2:**
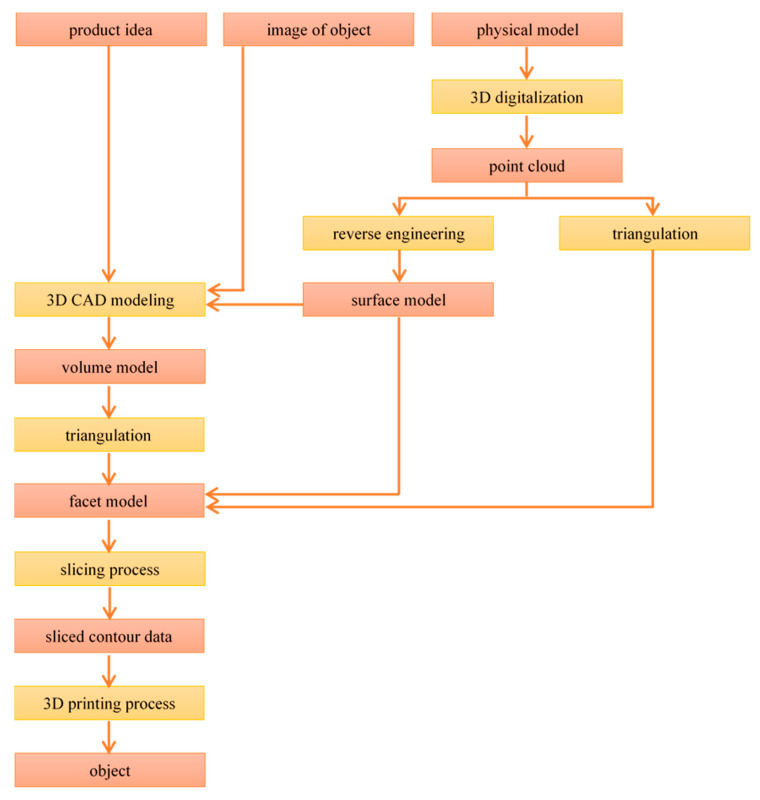
Stages of the additive manufacturing (AM) process.

Production of physical objects by 3D printing is a process of several stages. However, the individual steps are identical in most AM technologies (Figure 2).

The actions which AM includes can be divided into data preparation operations (building of a digital model, conversion of the model to the STL (Stereolithography) format, model verification, and preparation of the printing instruction file) and the operations of producing the physical object (its 3D printing and post-processing) [1,12].

#### 2.1.1. Preparation of Object Data for 3D Printing

**Figure 3:**
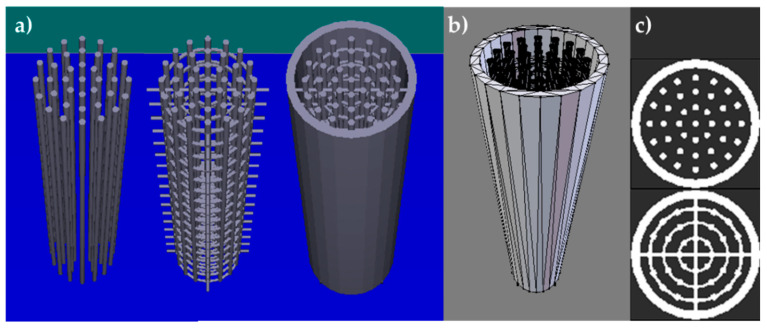
(**a**) Stages of designing 3D digital model (from left); (**b**) tessellated model; (**c**) slices (layers) into which the model is split.

The first stage is usually the development of a digital model of the object (Figure 3a). The digital model can be generated in a three-dimensional computer-aided design (3D CAD) system. It is done by geometric modeling, which includes surface and solid modeling. There are many 3D CAD modeling software products commercially available, like AutoDesk, AutoCAD, SolidWorks, Creo Parametric, FreeCAD, Rhino, and SketchUp [12,13,14]. A digital model can be produced by reverse engineering the geometric data gathered from a physical object. Physical objects can be digitized in 3D by touch-probe technology, laser scanning, or medical imaging, like computed tomography and magnetic resonance imaging. Any of these methods produces a point cloud from the physical object. The points in the cloud must be connected with a suitable software tool. Yet another method of 3D CAD modeling is the application of 2D capture geometry inside the technology. A combination of the methods can also be used to generate a digital model [1,15].

The second step is to generate a facet model by converting the digital model file into a suitable format, and STL is the most common one [1]. STL stands for Standard Tessellation Language or STereoLithography. An STL file contains information about every surface of the 3D model as triangular sections. The vertices of each triangle are defined with Cartesian coordinates and arranged in a text format. The number of triangles determines the resolution of the 3D model. An approximation of the digital model surface represented by triangles is triangulation (or tessellation) (Figure 3b). If written in this format, the data can be transferred from CAD software to a 3D printer. The geometric data produced from a physical object can be directly converted to the STL format [12,13,14]. STL can be replaced with another format, like the new international standard format called AMF (Additive Manufacturing Format), introduced by ASTM International [1].

The third stage is to generate cross-sectional data, which are instructions for the AM machine on how to print the object [14]. The 3D printer firmware interprets the data taken from one or more STL files to enable their preview and editing, which includes resizing, repositioning, reorientation, addition of supports; this affects the accuracy of the printed object, its surface finish, and the time to build and finish the object [1,13,16,17]. Some 3D printing technologies require supports to stabilize the entire object being built, including its overhangs, and to align temporarily unbound object parts, ensure attachment to the build platform, or prevent deformation of the object [18]. The object(s) with the supports are then mathematically divided (sliced) into a number of parallel horizontal planes with a dedicated firmware algorithm of the 3D printer. The space between each two successive horizontal planes of a definite and small thickness (usually within 25–100 μm depending on the 3D printing technology) and a defined contour is called a slice (Figure 3c). The output file (“G-code” file) is specific to the 3D printer brand and model. It is also possible to directly slice the 3D CAD model. Each slice is a single layer in the 3D printing process. The layers become “the tool paths” that drive a laser, a print head, or an extrusion tip, with which the model is printed onto an object [12,13,14,16,19].

#### 2.1.2. Building a Physical Object

Before an object is 3D-printed, it is necessary to set up the 3D printer by defining the build parameters and the physical preparation for the 3D printing cycle, which includes loading of the material, placement and leveling of the build platform, and preheating the 3D printing system [1,16]. Three-dimensional printing is an automatic process. An object is built along the Z axis defined in the STL models and from bottom up. A single layer is built with a defined thickness and a defined pattern and attached to the build platform. The second layer is built next and bound to the previous one. The sequence is repeated until the object is completely built. The layers can be built and bonded in many ways, including polymerization, melting and subsequent solidification, fusing, bonding, or extrusion. The layers can be manufactured from different materials, including polymers, ceramics, metals, or paper, and supplied in various forms: liquid, powdered, in filaments, or in sheets [17,18,20].

Once the 3D printing process ends, the object is separated from the build platform and processed to become finished. The object often requires cleaning by removal of excess material, which may include natural supports or uncured resin, and synthetic supports. An excess of powder can be removed by brushing, with jets of compressed air, by vacuum cleaning, application of vibration, with special tooling, or by immersion in a suitable solvent. Uncured resin can be removed by washing with a suitable solvent. Synthetic supports made of a secondary material can be melted away, dissolved with solvents, or removed by pyrolysis. Synthetic supports made of the primary material can be torn off or broken off by hand or with cutting tools. The appearance of the 3D-printed surface can be improved by sand blasting, shot peening, grinding, or polishing. Some 3D printing methods require specific post-building treatment, like drying, UV or VIS-light curing, coating, sintering, or infiltration [1,16,21,22].

### 2.2. 3D Printing Methods

There are many proposals for AM process classification [1]. Already in the 1990s, AM was proposed to be classified by the type of processed stock material, the layer building method (1D or 2D), and the printing technology [3]. Recently, ASTM International offered a new AM classification. The AM methods were grouped by the type of building and bonding of the layers of the material. Seven categories of AM method were defined: Vat Photopolymerization (VP), Powder Bed Fusion (PBF), Material Jetting (MJ), Binder Jetting (BJ), Material Extrusion (ME), Sheet Lamination (SL), and Directed Energy Deposition (DED) [2]. The short characteristics of each AM method are shown in Table 1. A detailed characterization of the AM (3D printing) methods is specified in multiple overview studies [10,23,24,25,26,27,28].

For the production of catalysts, researchers proposed to apply 3D printers which mainly operated by Material Extrusion (ME), in this way, they 3D-printed catalytic supports or finished catalysts [24,25]. Although inexpensive and simple, the methods do not provide satisfactory resolution, accuracy, or surface finish of 3D-printed objects [29,32]. Technologies were suggested recently which would provide better results, like VP: Stereolithography and Digital Light Processing [26]. Other methods potentially viable for building catalysts include Selective Laser Sintering, Selective Laser Melting, Binder Jetting, and Laminated Object Manufacturing [11,24,25].

#### 2.2.1. Material Extrusion Methods (ME)

The 3D printing methods based on Material Extrusion date back to the end of the 1980s, which is when Scott Crump developed Fused Deposition Modeling [33].

The ME technologies are based on the processes of forcing out (extrusion) of a semi-solid material formed into a thin filament of softened or molten thermoplastic polymer, a paste, a solution, or a polymer dispersion, under the pressure applied. The building material is deposited on a substrate and solidified in a predefined shape, by which the material is bound to the substrate or a previously extruded material [1,22,30,34]. Solidification can be achieved by lowering the temperature of the material, which takes place with materials preheated to a temperature of their melting point or glass transition (in semi-crystalline polymers and in amorphous polymers, respectively) before extrusion. These processes require a certain amount of residual heat to enable the bonding of adjacent surfaces. Solidification can also be triggered by chemical changes caused by a curing agent, a residual solvent, a reaction with atmospheric oxygen, or by drying of the material while it is wet. If the material is a gel or a paste, a residual solvent or a wetting agent must be present to ensure bonding of the new material deposition with the material already deposited [1,34].

In ME technologies, the building materials can be supplied in different ways. The materials to be liquefied are fed in the form of filaments on the spools into a preheated chamber. Commercially available filaments are produced by extrusion of a molten main thermoplastic polymer combined with fillers, fibers, pigments, and other additives. If the material is a granulate, it is fed into a screw extruded. Liquid materials, like pastes and solutions, are fed into a dedicated container (a cartridge). The extrusion process is performed by controlled application of force by a step motor, a piston, a pneumatic device, or an actuating roller. Having achieved the suitable form, the materials is extruded via a nozzle or an aperture of the extruder head which moves along a horizontal plane to deposit the layers of the physical object. In parallel with the layers of an object, supports can be extruded from the same material—or a different one, if the extrusion head is provided with more than one nozzle. With the whole layer deposited and depending on the design of the 3D design, the build platform descends or the printing head lifts by the height equal to the thickness of the layer, after which the deposition of the next layer begins. The sequence is repeated until the object is completely built. Once the 3D print run is complete and the object is removed, the supports are removed mechanically or with a suitable solvent, if made from a material different than the building material of the object [1,22,34,35,36].

**Figure 4:**
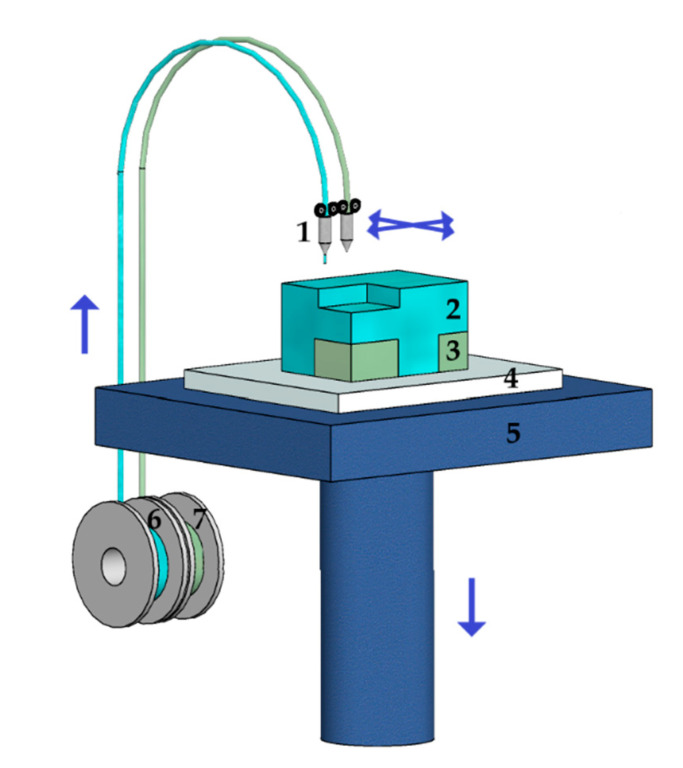
Fused Deposition Modeling as an Material Extrusion (ME) technology example: 1—extrusion nozzles, 2—part, 3—part supports, 4—base, 5—build platform, 6—build material spool, 7—support material spool.

The most popular ME method is Fused Deposition Modeling (Figure 4). A derivative of the technology is Fused Filament Fabrication. Both are based on the extrusion of a liquefied polymer fed to the printing system as a filament and its solidification by cooling [1,35,37]. The methods use the following polymers: acrylonitrile-butadiene-styrene, acrylonitrile-styrene-acrylate, Nylon-12, polycarbonate, polyetherimide, poly(lactic acid), thermoplastic polyurethane, poly(vinyl alcohol), poly(ethylene terephthalate), and thermoplastic elastomers [29,38].

A modification of Fused Deposition Modeling exists for building ceramic objects. Fused Deposition of Ceramics is the extrusion of a liquid ceramic/polymer suspension, where the ceramic material is usually 40–45% of the binder volume [36,39]. Robocasting is a process which uses aqueous suspensions, colloid gels, and ceramic or composite pastes. The process is nearly binderless. The solidification occurs by evaporation of the solvent. The 3D-printed objects are dried and sintered [30,36,40]. Robocasting is also known as Robotic Deposition, as well as Direct Writing, Direct Ink Writing, and 3D Fiber Deposition [8]. A similar technology is called Freeze-Form Extrusion Fabrication; here, however, post-extrusion solidification occurs by freezing the deposited material [40].

Moreover, 3D Dispensing (3D Plotting and 3D Micro Extrusion) is a group of methods where solidification can be triggered physically or chemically. The physical triggers may include crystallization and glass transition of thermoplastic materials, coagulation of polymer dispersions, and drying and precipitation of polymer solutions. The chemical reactions which are viable triggers may include cross-linking of thermosetting reactive pre-polymers and formation of ionomers and complexes of polyelectrolytes. Another solution is 3D printing of polymers in liquid media, zero-gravity printing, reactive printing, or 3D bioplotting. Initiators, activators, co-reactive resins, curing agents, or metallic salts are added to a liquid medium to prompt solidification by triggering an instant chemical reaction. Three-dimensional dispensing printing allows the deposition of many classes of materials, including thermoset resins like epoxides, acrylics, silicones, polyurethanes; metals and oxides thereof; ceramic materials like calcium phosphate, silicates, and bentonite; and biopolymers and hydrogels [34].

ME printing methods provide many benefits. Fused Deposition Modeling provides simple processing, relatively low-cost machines, a multitude of inexpensive and non-toxic stock materials, and printing with more than one material simultaneously. Fused Deposition Modeling has several drawbacks, like relatively poor accuracy and rough surfaces. A typical XY-plane resolution of a printer is 400 μm only, which is dependent on the nozzle orifice diameter. The objects are prone to develop structural defects and reduced mechanical strength; the mechanical performance often reveals anisotropic effects. The printing process is protracted if the Z axis resolution is high (for lower layer thickness) [1,30,31,35,41].

#### 2.2.2. Vat Photopolymerization Methods (VP)

Vat Photopolymerization methods are often termed stereolithographic, because they originate from Stereolithography, a technology invented in the 1980s by Charles Hull [42,43]. This technology comprises the curing (solidification) of a photosensitive liquid and multifunctional prepolymer (resin) in the presence of photoinitiators and light radiation, which supplies the energy to trigger a chemical chain reaction of polymerization. This process results in the binding of a large quantity of small molecules, which form a highly cross-linked polymer which is non-melting and non-soluble [44].

The stereolithographic methods use acrylic resins, methacrylate resins, epoxy resins, vinyl ethers, and ceramic or metallic powders suspended in the resin medium. Aside from a monomer or an oligomer, the resins include diluents, chain transfer agents, photoinitiators, and additives [42,44].

VP 3D printing is done in a resin-filled vat in which the build platform is immersed. The printing pattern is displayed on the resin surface. By exposure to light radiation, the resin solidifies in the pattern and down to a defined depth as a set of elementary volumes called voxels. This is when the resin is bonded to the build platform. Next, the platform is repositioned and the built layer is coated with liquid resin. The pattern of the next layer is displayed; the resin solidifies and bonds to the previous layer. It happens so because the curing depth is slightly more than the distance by which the build platform travels along the Z axis. Both steps, the shift of the build platform and the curing of the preset pattern in the layer of resin, are repeated in sequence until the finished object is built [44,45].

Depending on how the energy input is delivered (how the process is initiated), two classes of process are defined: single and two-photon. The single-photon processes include conventional stereolithography, where UV light and photosensitive resins are used, IR stereolithography, where IR light and thermosetting resins are used, stereo-thermal-lithography, where UV and IR light is used to build multi-material structures, and a VIS-light printing method. The two-photon absorption method is applied to build micro- and nanoscale structures within the volume of the resin. It applies femtosecond laser light pulses [44].

Conventional single-photon methods are divided into two approaches: direct or laser writing, which is a vector scanning with the laser beam, and mask-based writing, which is irradiation of the entire photopolymer-filled vat surface with a flood lamp and a dynamic mask applied, done most often with a digital micromirror device (DMD). A DMD is an array of micro-sized mirrors which can be rotated into active or inactive position. This allows the light to be reflected only from a specific portion of the array (the active mirrors) to reproduce a defined image [44,46].

**Figure 5:**
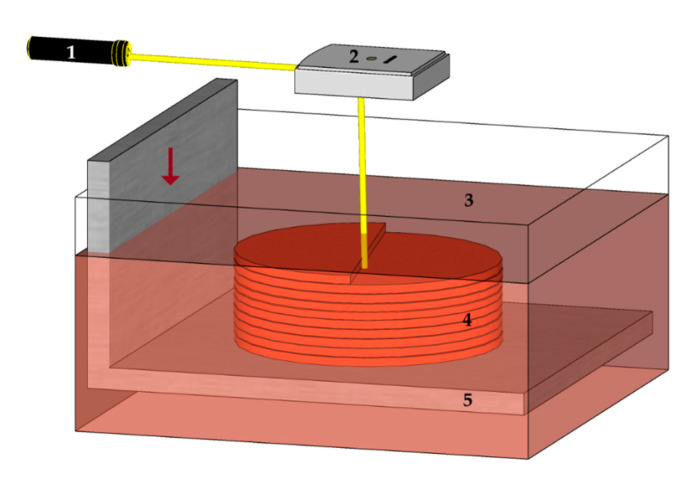
Vat Photopolymerization-based Stereolithography: 1—laser, 2—scanner, 3—vat of resin, 4—part (cured resin), 5—stage.

**Figure 6:**
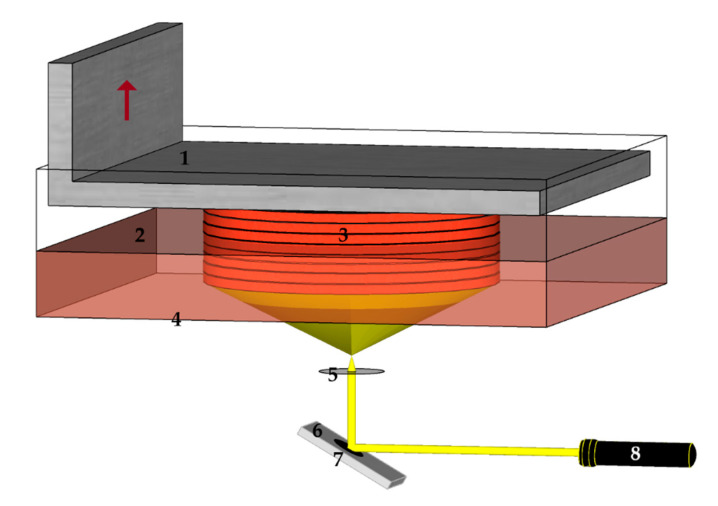
Vat Photopolymerization-based Digital Light Processing: 1—stage, 2—vat of resin, 3—part (cured resin), 4—transparent window, 5—lens, 6—digital mirror device, 7—pattern, 8—laser.

VP printing machines may vary in configuration, i.e., the motion of the build platform. In the bottom-up method, the build platform is just below the surface of the resin. An exposed thin layer of the resin is irradiated from the top, by which it is cured on top of the structure. The build platform moves down by a defined step and thus it is flooded with a layer of fresh resin. The top-down method is becoming popular. In this case, the build platform is immersed in the resin from the top, leaving only a thin layer of resin between the build platform and the vat bottom. The resin is irradiated from the vat bottom, which is a transparent non-adhering plate. With the irradiated resin layer cured, the build platform goes up by a defined step and the liquid volume of the resin in the vat fills the gap between the build platform and the vat bottom [42,45]. Conventional Stereolithography uses the bottom-up approach and laser radiation (Figure 5). Digital Light Processing uses the top-down method and projector light (Figure 6). Although the structure being built top-down is exposed to higher mechanical forces because it must be separated from the bottom plate once the layer is irradiated, this approach has several advantages over the bottom-up systems. The object does not require recoating, the irradiated surface is always smooth and only small volumes of resin are required for each layer, and the irradiated layers are not exposed to the atmosphere, which reduces oxygen inhibition and the overall 3D printing cycle duration [45].

Once the printed structure is cleaned of surplus uncured resin and the supports are removed (if any), the workpiece is most often cured with UV light to achieve full conversion of the reactive chemical groups and improve the mechanical performance [18,45].

Yet another version of VP is Continuous Liquid Interface Production. This method works by photopolymerization, but the resin is cured continuously. Here, the 3D printers resemble the Digital Light Processing printers [34]. The innovative feature is the dead zone, a thin layer of uncured resin between the printed workpiece and the vat bottom. The dead zone is formed by application of a UV-transparent, oxygen-passing window in the base of the vat. Below the window, a constant supply of pure oxygen is provided. The UV transparency of the window allows the laser beam to penetrate the resin vat and cure the resin, while permeation by oxygen allows the gas to penetrate the resin vat to inhibit polymerization. The dead zone ensures a constantly fresh layer of resin under the object being printed. The build platform moves continuously [42].

An interesting area of AM is ceramic Stereolithography. It is a method of additive manufacturing of high-quality ceramic objects by photo-cross-linking a resin material loaded with ceramic powder. Following the building process, the binder is burned away and the part is sintered. Unlike injection molding of ceramic objects, stereolithography allows the production of structures with much more complex geometric features with reduced cost and time when compared to the production of ceramic objects with matrices [25,47].

The drawbacks of VP technologies include applicability to a limited choice of materials, high costs of processing equipment, chemical waste, the need for finish processing, built object shrinkage, a compromise between high throughput and high resolution, and not perfectly smooth object surfaces. The 3D printing process is relatively slow because of the slow photopolymerization process and the multi-stage mechanism of building. It is not so with Continuous Liquid Interface Production, which is the fastest VP method since it does not include a resin coating step. An advantage of stereolithographic methods is the high resolution of objects. Irradiation by a projector helps to achieve higher resolution, defined by the pixel size, not by the spot size of the laser beam. It also provides the higher working speed, since the whole area of each layer is irradiated at the same time. The typical resolution ranges of the systems are 20–100 μm in Digital Light Processing, 50–100 μm in Stereolithography, and 75 μm in Continuous Liquid Interface Production [34,42,45].

#### 2.2.3. Other Methods

Powder bed fusion is a process in which thermal energy is applied to selectively fuse areas of a powder bed. Thermal sources such as lasers and electron beams are used to induce the fusion of powder particles. Most processes utilize the following fusion mechanisms: liquid-phase sintering (metals, composites) and full melting (metals, polymers). Solid-state sintering and chemically induced binding (ceramics) are also possible [1].

In the process of Selective Laser Sintering, the powder is spread on a built platform by a roller. The laser beam scans the preheated powder selectively and sinters the powder particles based on 3D CAD data to form the slice cross-section. The surrounding loose powder is a support. Then, the building platform is lowered by one layer thickness and the next layer of the powder is spread, leveled, and scanned by the laser beam. The process repeats until the complete object is printed. The process of Selective Laser Melting is similar. Instead of sintering, alloy powder particles are melted and resolidified. Electron Beam Melting uses the focused electron beam for melting metals and alloys powders in a vacuum chamber at high temperature [48].

Material Jetting involves processes in which droplets of a liquid material are deposited selectively and converted to a solid geometry. The droplets are dispensed in a continuous stream or a drop on demand mode. Printers use two single jets or print heads with many nozzles, like inkjet printers, in order to deposit layers of a building material and a support material. During the printing processes, the print head or the substrate moves, creating an object layer by layer. The phase change of the printed material usually takes place as a result of solidification of the melted material, e.g., thermoplastic polymer, wax, metal, which is cooled by giving off heat to the environment. This is typical of the Thermojet process. In addition, the curing of a photopolymer in the process of photoinitiated polymerization with UV light is used in the PolyJet and Multi-Jet technologies (e.g., Projet printer). The evaporation of the liquid part of a solution or slurry of the ceramic material and other chemical reactions are also possible [1].

The best resolution, accuracy, and finish quality of object surfaces can be produced with Vat Photopolymerization and Material Jetting technologies [29,32]. The least expensive 3D printing devices work by Material Extrusion. They work fast and are easy to operate [31,41]. All these features have driven the high interest of researchers in VP, MJ, and ME. For over a decade, 3D-printed structures have been researched, mainly those produced by ME, for their feasibility in various areas of chemical processing.

## 3. 3D Printing Applications in Heterogeneous Catalysis

Today, AM technologies are applied in many fields, like medicine, which includes dentistry and pharmacy, as well as food processing, automotive manufacturing, art, architecture, education, entertainment, engineering, automation, robotics, electronics, and the aerospace industry [22,32,35]. A great number of AM applications exist in chemistry and derivative fields, like electrochemistry [49], analytical chemistry [50], and biotechnology [51]. This work focuses on AM applications in the field of catalysis.

### 3.1. Directly Produced Structures

Three-dimensional printing coupled with CAD helps to develop unique monolithic structures which are otherwise impossible to produce with traditional methods [24]. Three-dimensional printing enables direct synthesis of monolithic catalyst supports and finished catalysts, static mixers, and more.

The catalytic active phase can be integrated with the monolithic structure already during 3D printing or deposited on a finished object. Many applications require finishing, like in metallic and oxide catalyst synthesis. The finishing process includes drying and sintering to remove the binder and decompose the precursors. Sometimes, the products require reduction of metal ions. In carbon-based material synthesis, finishing is required to carbonize carbon precursors, like thermosetting resins or starch [24].

In recent years, different types of 3D printers have been applied to produce monolithic structures from doped polymeric materials, carbon materials, metals, metal oxides, and zeolites. The catalytic performance of the materials has been tested in many processes, like hydrocarbon transformation [24].

#### 3.1.1. Monolithic Catalysts

##### Ceramic and Zeolitic Monoliths

- Al_2_O_3_ monoliths

The first monolithic structures built with 3D printers were ceramic support structures of aluminum oxide manufactured by a direct fabrication technique: Robocasting. They were first produced in 2003. The ceramic supports were sintered, following by coating with a layer of a hexaaluminate material, BaMn_2_Al_10_O_19-α_. Catalysts made entirely of hexaaluminate were also 3D-printed. Their catalytic activity was determined in a process of methane combustion. The robocast lattices of BaMn_2_Al_10_O_19-α_/Al_2_O_3_ converted approximately six times more methane at 600 °C than a cordierite honeycomb monolith, produced by conventional extrusion, with the same amount of active catalytic phase. The increase in the active phase content in the monolith improved the methane conversion to 45% at 600 °C. The robocast monolith of BaMn_2_Al_10_O_19-α_ provided methane conversion of 89% at 600 °C and 100% at 700 °C [52].

Robocasting was applied to build monolithic supports based on α-Al_2_O_3_ and coated with γ-Al_2_O_3_ and Pt. A specially designed geometry of the catalyst helped to achieve high conversion of carbon monoxide (at up to 100%) in oxidation reaction of high flow rate. The catalysts supported on commercially available cordierite honeycomb structures performed worse [53].

Robotic Deposition of an ink loaded with powdered Al_2_O_3_ and a Cu(II) salt, followed by sintering of the built object, produced a woodpile porous system with Cu in a matrix of Al_2_O_3_. The Cu/Al_2_O_3_ monolithic structures were characterized by excellent catalytic performance in Ullmann reactions (the synthesis of imidazoles, benzimidazoles, and N-aryl amides). The structures provided high yields of transformation into N-aryl compounds (78–94%) with a short reaction time (of 2 to 4 h). The great chemoselectivity of the transformation was noted, along with great recyclability; the systems recovered from the reaction were reusable at least in 10 more reactions without a high loss of yield [54].

A controlled-porosity monolith made from Al_2_O_3_ by Robotic Deposition was applied as a Lewis’ acid in the reactions of synthesis of 1,4-dihydropyridine and 3,4-dihydropyrimidin-2(1*H*)-one compounds. The 3D-printed catalysts provided remarkable efficacy, with extremely good yields in Biginelli and Hantzsch reactions with short reaction times under solvent free conditions (70–95% of yield over 30 min). The monolith was also recyclable and reusable up to 10 times without any loss of activity [55].

- SiO_2_ monoliths

Monolithic silica supports were produced by Robotic Deposition followed by sintering [56,57]. The support surface was modified by silanization and metalation to produce Pd/SiO_2_ and Cu/SiO_2_ catalysts. The performance of the catalysts was tested in bicatalytic heterogeneous reactions of transformation in solutions based on Cu-catalyzed azide−alkyne cycloaddition and Pd-catalyzed cross-coupling (the reactions of Sonogashir, Stille, and Suzuki). The applied monolithic structures enable rapid generation of substituted benzyl-1,2,3-triazoles. The catalysts demonstrated stable performance. They could be recycled and reused at least 10 times [56]. In another research project, the surfaces of 3D-printed silica supports were functionalized with a polyimide-palladium composite. The monolithic catalysts were applied as the first component in a tricatalytic system for multistep in-solution one-pot transformations. The remaining components of the tricatalytic system were ferritic Cu(I) magnetic nanoparticles and a 3D-printed (manufactured by Fused Deposition Modeling) polypropylene capsule-containing Cu(II) loaded onto polystyrene-supported triazabicyclo[4.4.0]-dec-5-ene. The system was tested in a sequence of the following reactions: Chan-Lam’s azidation, Cu-catalyzed alkyne-azide cycloaddition, and Suzuki reaction. The substituted 1,2,3-triazoles were produced with a high reaction efficiency and without any special additives or intermediate isolation. All tested catalysts were readily recovered and reused in multiple reaction cycles [57].

- Zeolite monoliths

Robocasting was applied in AM of ZSM-5 zeolite-based structured catalysts which vary in architecture. The structures were intended for MTO (methanol to olefins) conversion. As the 3D-printing filament diameter was decreased, the stability and activity of the resulting catalyst was improved in the MTO process; as the macroporosity of the monolithic structure increased, the stability increased while the catalytic activity was reduced. The modification of structural features slightly affected the selectivity. A ZSM-5 catalyst with a binder system of silica and aluminophosphate and featuring zig-zag channels in the direction of flow provided selectivity to C_2_-C_4_ olefins at up to 68.9% with a methanol conversion of 90% at 450 °C. The catalyst exhibited better activity and stability than a monolith with straight channels [58].

Another research team used a laboratory-scale 3D printer to extrude zeolite catalysts based on HZSM-5, HY, and ZSM-5 doped with various metal oxides [59,60,61,62,63,64]. An MTO process was performed to test the monoliths made of HZSM-5, including monoliths which also contained amorphous silica integrated with the structure and monoliths surface-coated with SAPO-34 zeolite. The application of 3D-printed catalysts instead of powdered catalysts improved the selectivity to light olefins. The ethylene to propylene ratio could be adjusted with the layers of SAPO-34. The highest methanol conversion which reached 100% was achieved with the HZSM-5 monolith coated with SAPO-34. The lowest conversion was shown by the HZSM-5/SiO_2_ monolith without SAPO-34. The tested monoliths demonstrated better stability than a powdered HZSM-5 catalyst [59]. An MTO process was also used to test a series of ZSM-5 monoliths doped with oxides of Ce, Cr, Cu, Ga, La, Mg, Y, and Zn. The addition of Cr, Mg, and Y to the monolith did not result in a significant drop in methanol conversion. The addition of Zn and Mg provided the best selectivity to light olefins. An extremely promising version was a monolithic Mg/ZSM-5 catalyst, which enabled selectivity to ethylene and propylene at 24% and 33%, respectively, with a methanol conversion of 95% at 400 °C and with a reduced amount of coke deposition [60]. Another series of ZSM-5 monolithic structures with oxides of Ga, Cr, Cu, Zn, Mo, and Y was tested in a process of methanol conversion to hydrocarbons in an atmosphere of nitrogen and carbon dioxide. The yield of light olefins was enhanced over all doped monoliths used in the N_2_ atmosphere. With N_2_ replaced with CO_2_ and with the reaction maintained at 400 °C, the selectivity to ethylene decreased, while the selectivity to propylene was almost constant. The Y and Zn-doped monoliths proved a higher selectivity of light olefins and BTX compounds (benzene, toluene, and xylene) in the absence and presence of CO_2_, respectively [61]. The catalytic performance of the monolithic structures 3D-printed with HZSM-5 and HY zeolites, including a version with the surface modified by SAPO-34, was also tested by n-hexane cracking. The HZSM-5 monolith had more stable activity and higher selectivity to light olefins than its powdered counterpart, with the highest selectivity of 53.0% determined at 650 °C. The HY monolith could produce light olefins with a selectivity of 57.9% at 600 °C. The addition of the SAPO-34 increased the activity in all tested monoliths and markedly improved the selectivity to BTX in comparison to the HY monoliths. The selectivity was 27.5% for the SAPO-34-coated HY monolith catalyzing at 600 °C [62]. The ZSM-5 monoliths with the matrix laden with Cr, Cu, and Ni provided high selectivity to BTX in the n-hexane cracking process, whereas the ZSM-5 monolith doped with Y provided higher selectivity to light olefins. The temperature and reaction time significantly changed the distribution of the reaction products. The maximum selectivity to light olefins was achieved at approx. 50% by running the reaction with the ZSM-5 monolith doped with Y [63]. Structured monoliths with hierarchical porosity and controlled type and density of acid sites, such as HZSM-5 with or without a SAPO-34 layer on the surface, were obtained. The catalytic performance in the conversion of methanol to dimethyl ether indicated that the selectivity toward dimethyl ether was favored by the HZSM-5 monolith (DME (dimethyl ether selectivity) of 96%, methanol conversion of 70% at 180 °C) compared to the powder catalyst and the HZSM5@SAPO-34 monolith. The SAPO-34 growth resulted in further conversion to higher hydrocarbons [64].

- TiO_2_ monoliths

Monolithic structures were manufactured by the Robocasting technique from a paste of titanium dioxide nanoparticles in an acidic medium. These structures were processed next by low-temperature chemical sintering. The TiO_2_ monoliths developed high photocatalytic activity in an air purification reaction of decomposition of acetaldehyde to CO_2_ and H_2_O. The concentration of removed acetaldehyde varied with its amount in the gas. The yield for 5000 ppmv acetaldehyde was 40–58%, while for 70,000 ppmv, the removal yield was approx. 8% [65].

Au/TiO_2_ monolithic catalysts were prepared using a technique similar to Fused Deposition Modeling from a paste containing TiO_2_ and nanoparticles of Au, or by deposition of Au on the ready-printed TiO_2_ monolith. In the process of hydrogen photoproduction from a water and ethanol mixture in the gaseous phase, the monoliths impregnated with Au after 3D printing (so-called post-impregnated) provided higher efficiency—here, the monoliths had a total concentration of Au 100 times lower than the monoliths 3D-printed from the Au-doped paste, while the amount of Au on the surface of the microfilaments was similar in both monolith versions. The rates of hydrogen photoproduction for the post-impregnated monoliths were 2 to 3 orders of magnitude higher. The lower the monolith filament diameter was, the higher the efficiency of hydrogen photoproduction was. The best photoactivity, determined at 0.24 mol H_2_ min^−1^ g_Au_^−1^, was achieved with the post-impregnated titanium monolith 3D-printed with filaments 200 μm in diameter [66].

- Other monoliths

Silicon carbide monolithic catalysts doped with nanoparticles of iron were built by Robocasting and post-treated at a high temperature. In the wet peroxide oxidation of phenol, the Fe/SiC monoliths provided good catalytic activity, high efficiency of H_2_O_2_ decomposition, and long-term stability (350 h) [67].

PtO_2_-WO_3_ catalysts with complex shapes were manufactured via Digital Light Processing from a solution composed of resin and metal salts. The objects were then pyrolyzed to produce oxides. The 3D catalysts were tested for the hydrogenation of alkynes and nitrobenzene and showed excellent activity in these catalytic reactions, i.e., in the hydrogenation of phenylacetylene, the conversion was full after 6 h, while the selectivity towards styrene was 82% [68].

##### Metallic Monoliths

The method of three-dimensional fiber deposition was applied to print monoliths by extrusion from a paste loaded with powdered metallic alloys. Once sintered, the monoliths were used as supports of catalysts for different reactions [69,70,71]. Structures of Ti6Al4V alloy were alkali-treated and coated with a layer of ZSM-5 zeolite. A dope of Fe was used to gain catalytic activity in a reaction of nitrous oxide decomposition. The catalyst doped with 10% of Fe revealed the highest catalytic activity with stable performance. Over 160 h of the process at 600 °C, the loss of conversion was less than 5%. A proper geometry of the monolith was developed which improved the N_2_O conversion at the processing temperature (with up to 100% at 800 °C) [69]. Monoliths made from 316L stainless-steel were coated with layers of ZSM-5 combined with silica. The porous catalysts were tested in a methanol-to-olefin conversion process. At 250 °C, high selectivity to dimethyl ether was achieved with a catalyst which featured straight channels. However, in the process run at 350 °C, structured catalysts were effective in converting methanol to olefins even at high feed rates of methanol. The monolith with tortuous channels provided the highest yield of light olefins, reaching approximately 40%. The methanol conversion in the process run with structural catalysts was higher than with a packed bed [70]. The catalytic performance of 316L stainless-steel monolithic structures coated with Ni/Al_2_O_3_ catalyst was tested in a reaction of carbon dioxide methanation. In comparison to a conventional powdered Ni/Al_2_O_3_ catalyst, the monolithic catalysts provided higher CO_2_ conversion, especially at high temperatures, like 90% at above 370 °C. In addition, the monoliths exhibited higher stability; in the presence of one of these monoliths, the process run for 53 h at 350 °C had stable conversion at approx. 80%. The best results were achieved with a zigzag architecture 3D catalyst, which gave CO_2_ conversion of 91% and selectivity to CH_4_ of 98% at 400 °C [71].

The composite inks loaded with gold and silver were used to print 3D structures by direct ink writing. The alloy structures formed by processing at a high temperature were immersed in a nitric acid bath to remove Ag. This produced monolithic nanoporous gold. The catalysts were tested in a reaction of selective partial oxidation of methanol to methyl formate and CO_2_ at a high temperature. The 3D-printed structures had selectivity to methyl formate (70–90%) comparable to that of the Au nanoparticles. While comparing the reaction rate per the catalyst mass, the 3D-printed catalyst outperformed nanoparticles by two times for this metric [72].

##### Carbon Monoliths

Carbon monoliths were produced by 3D printing of carbon source materials (doped with additives) by extrusion followed by pyrolysis (carbonization) in an inert gas shield of nitrogen [73,74]. By Solid Free Forming with an ink loaded with metal precursors, poly(vinyl alcohol), and starch, the Ni and Mo-doped carbon structures were developed. The carbon scaffold contained up to 25 wt.% of the catalyst material. A reaction of syngas conversion to higher alcohols was performed. At high flow rates of the syngas feed (6000 h^−1^), the CO conversion dropped quickly to 16% with pelleted catalysts, while the structured catalysts converted 35% of the CO [73].

Direct Ink Writing was applied to print monoliths from ink loaded with starch, gelatine, and SiO_2_ as a hard template. Following a process of carbonization and template removal, the carbon monoliths were applied to catalyze the liquid-phase selective oxidation of benzyl alcohol. The monolithic structure had a significant impact on the reaction rate. High conversion was achieved with high selectivity to benzaldehyde [74].

##### Polymeric Monoliths

Polymeric monolithic structures were obtained by 3D printing with the use of photosensitive liquid resin mixed with carbon or silica. The active CuO/CeO_2_ phase was then deposited by dip coating. Modification of the channel wall design was necessary to anchor a large amount of the active phase. Thermal treatment in air was needed to recover the activity of the active phase. The monoliths demonstrated good catalytic activity, stability, and reusability in the preferential oxidation of CO in the presence of O_2_ and H_2_ with He balance. The maximum CO conversion was 97% at 150 °C (slightly lower than that of the powdered catalyst), with a temperature delay of 25 °C. After several reuse cycles, the activity of the supported catalyst increased [75].

Devices of well-defined shapes, including woodpile and holed structures, were designed and synthesized using thiourea-embedded resin and a stereolithography 3D printer. These structures were then used to catalyze the addition of N–Me–indole to trans-β-nitrostyrene (Friedel–Crafts alkylation). The printed organocatalytic materials promoted the formation of the desired product with a yield up to 79%, but the reaction times were longer than in homogeneous processes [76].

Three-dimensional polymeric structures have found applications in biocatalysis. Catalytically active living materials being composites of live yeast cells in a polymer matrix of F127-dimethacrylate were 3D-printed by direct writing and underwent photochemical cross-linking. The produced cubic structures demonstrated metabolic activity during fermentation of glucose. Ethanol was produced with a yield of approx. 90%. No significant reduction in catalytic activity was found over 2 weeks of batch processing [77].

#### 3.1.2. Static Mixers

Structural catalysts include monoliths and static mixers. Static mixers are open cross-flow structures characterized by intense radial mixing. They provide high-efficiency transport of mass and heat through the whole cross-section and even in conditions of laminar flow. The very narrow residence time distribution makes the flow pattern close to the plug-flow. Given the twisted flow path of reactants in the structures, the pressure drop is higher than in monoliths of the same voidage but remains relatively low [78].

Researchers designed and produced a 3D structure by Selective Laser Sintering. The structure was a combination of a catalyst carrier (a static mixer) and a reactor, forming a porous structured reactor. The carrier was coated with a layer of Al_2_O_3_ and ZnO, and Pd nanoparticles. The porous structured reactor applied instead of a conventional batch reactor in a process of solvent-free selective hydrogenation of 2-methyl-3-butyn-2-ol to 2-methyl-3-buten-2-ol resulted in slightly improved selectivity (up to 97.6%) and yield (up to 97.3%) [79].

Another research team proposed manufacturing of static mixers by Electron Beam Melting from the alloys of Ti6Al4V, CoCr [80], 316L stainless-steel [81,82,83]. Layers of catalytically active metals were deposited on the static mixers. Various catalytic systems of the static mixers were fitted inside steel tubular flow reactors [80,81,82,83]. The catalytic mixers coated with Pt and Ni were tested in a series of processes of alkenes and carbonyl hydrogenation. The increase in reactor pressure in the vinyl acetate hydrogenation reaction with Pt/Ti6Al4V and Ni/CoCr catalysts improved the conversion. The Pt catalyst brought the conversion to 83.2–92.1% at 20–24 bar. A high conversion of cinnamaldehyde (88.7%) was achieved in reaction with a Pt catalyst [80]. The steel systems coated with a Pd or Ni layer were tested in the hydrogenation of alkenes, alkynes, carbonyls, nitrogen compounds, nitriles, imines, and halides. Hydrogenation by feeding gaseous H_2_ had conversion above 75% in most reactions, and in several cases, the conversion was full. It was found that, in some cases, the hydrogenation selectivity could be influenced by modifying the operating parameters of the reactor. Depending on the actual pressure and flow rate in the reactor with a Ni static mixer, phenylacetylene was hydrogenated to styrene or ethylbenzene. A similar phenomenon was found during hydrogenation of cinnamaldehyde in reaction with a Pd static mixer [81]. The systems coated with Pd and Ni were tested in reductive amination of aldehydes and ketones (i.e., functional amine synthesis). Usually, only one synthesis product was formed without any side products with a high conversion (>90%). In those processing runs where the intermediate compound formation stage was slow, a two-step procedure was applied which included an additional flow reactor to increase the total conversion. For example, when the synthesis of N-benzylaniline was switched to the two-step procedure with a Pd catalyst, the conversion was improved from 12% to 84% [82]. Pd static mixers were also applied in the production of an intermediate compound for an antimicrobial drug, linezolid, by reduction of substituted nitrobenzene to a corresponding amine. This hydrogenation process helped with the production of the chemical compound at a yield of 0.5 kg a day, which is three times the output of the current flow reaction methods. Another advantage was the lack of necessary removal or recovery of the catalyst [83].

For processes in a batch reactor, the following solution was proposed: a mixture of Pd/SiO_2_ and polypropylene powders was processed by Selective Laser Sintering to build 3D porous catalytic objects intended to work as magnetic stir bar covers. In hydrogenation of styrene and phenylacetylene, the catalytic activity of the magnetic stir bar covers was similar to powdered Pd/SiO_2_ catalysts, but they did not perform as well in hydrogenation of cyclohexene [84].

#### 3.1.3. Other Structures for Catalytic Applications and Similar Fields

Catalysts are usually produced by binding a catalytic material somehow with the catalyst support; it is also possible to bind catalytic materials with the inner surfaces of reaction vessels (reactionware) with complex geometric features. Various 3D printing technologies are used for the production of reactionware. Two approaches exist: integration or functionalization. Integration involves including the catalyst species within a valid build material before the 3D printing process or using it as the building material outright. Functionalization consists in coating a 3D-printed structure with an active catalytic phase at the stage of 3D print postprocessing [8]. The application of 3D printing helps to build batch reactors and flow reactors. Aside from application in chemical synthesis, fluidic devices are often used in the chemical analysis of small volumes of substances and called micro- or millifluidics [85].

A system was developed in which a catalyst was 3D-printed into the structure of a reactionware piece made from an acetoxysilicone material. It was done by Robocasting an acetoxysilicone polymer paste doped with Pd/C. The catalyst was tested in hydrogenation of styrene to ethylbenzene, where the hydrogen source was Et_3_SiH. Quantitative conversion of styrene was observed in 30 min of the reaction at room temperature [86].

An integrated reactionware was manufactured and complete with reagents, catalysts, and purification apparatus for performing the following reactions: Diels–Alder cyclization, imine formation, and imine hydrogenation to a corresponding secondary amine. The base of the reactor structure was 3D-printed by Fused Filament Fabrication from polypropylene. The catalyst components were printed over by Robotic Deposition at specific locations of the structure. Acetoxysilicone polymer doped with montmorillonite K10 or Pd/C was used as the building material. The yields of the compounds were slightly lower than those of the compounds synthesized in standard laboratory glassware [87].

A number of 2D and 3D structures were printed for application in photochemical processes. Thin films of TiO_2_ were printed with a modified office inkjet printer on glass plates. The photocatalytic activity was confirmed in the reactions of decomposition of 2,6-dichloroindophenol [88], methyl orange [89], and methylene blue [90,91]. The active films for the third reaction were also produced by Robotic Deposition of hybrid inks doped with TiO_2_ [92]. Three-dimensional fiber networks of Al_2_O_3_ ink were also produced by robotic printing. The structures were cured with UV light and sintered. When a layer of TiO_2_ nanoparticles was deposited on the ceramic networks, they demonstrated photocatalytic activity in the reaction of formaldehyde decomposition similar to that of powdered TiO_2_ catalyst [93]. The mesh-form catalysts were also produced by Fused Deposition Modeling from low density polyethylene filaments with deposited TiO_2_. These catalysts were applied as floating photocatalysts in the degradation of organic pollutants in wastewater and they were found to be efficient in the removal of ofloxacin [94]. Porous 3D structures were built by indirect inkjet printing with CaSO_4_. Once impregnated with SiO_2_ and TiO_2_, the structures were tested in a process of degradation of wastewater pollutants. It was demonstrated that the photocatalysts helped to achieve more than 50% and nearly 90% of methylene blue conversion over 1 h and 5 h of irradiation, respectively [95]. Structures of various shapes built by Fused Filament Fabrication from polymer nanocomposites, i.e., ABS (acrylonitrile-butadiene-styrene) doped with TiO_2_, proved to be efficient photocatalysts in the degradation of rhodamine 6G [96].

AM technologies were used to make 3D catalytic structures for electrochemical processes, like electrolysis of water [8]. Steel structures of various designs were built by Selective Laser Melting [97,98,99]. Oxygen was generated with IrO_2_ [97,98] or NiFe [99]-coated structures. Hydrogen was generated with Pt, Ni [98], or Ni-MoS_2_ [99]-coated structures. Fused Deposition Modeling was applied to build carbon electrodes of graphene and poly(lactic acid). To make them useful in the production of hydrogen gas, they were activated [100] and coated with MoS_2_ [101].

Researchers 3D-printed many structures from materials which, once sintered, developed properties potentially significant to catalyst applications, like cellular structures built by bioplotting with metallic or oxide forms of Fe and Ni [102] or periodic structures based on ZnO and built by Robocasting [103].

### 3.2. Indirectly Produced Structures

Another solution proposed was to apply 3D printing in the indirect synthesis of catalysts, with the use of printed matrices (molds) [104]. This method is called “casting” and is used in the synthesis of ceramic materials mainly for medical applications. Polymeric matrices are built by Fused Deposition Modeling, Stereolithography, or Thermojetting, filled with ceramic material, and sintered to burn away the matrix [105].

**Figure 7:**
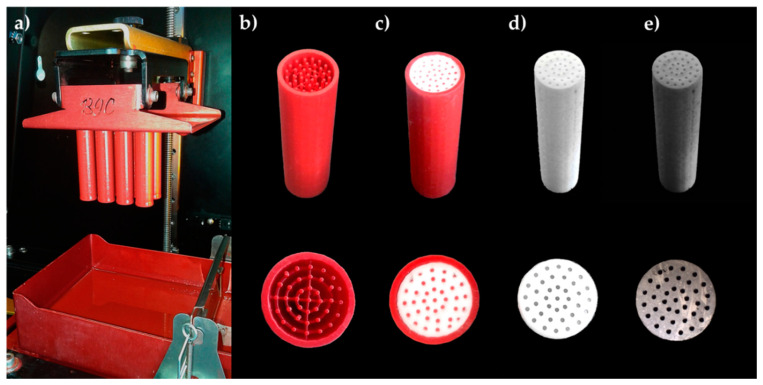
Application of 3D printing in indirect production of monolithic catalysts: (**a**,**b**) 3D-printed polymer matrices; (**c**) a matrix filled with ceramic material; (**d**) a catalytic support after removing the matrix; (**e**) a catalyst with the deposited active phase.

The indirect production of monolithic catalysts by the application of 3D printing also consists in replication of the 3D-printed matrices (templates) (Figure 7). Templates were printed with a polymer material on a high-resolution 3D printer which used Digital Light Processing. The cleaned and cured templates were filled with a paste of corundum (α-Al_2_O_3_) and sodium silicate solution; in some versions, other active components were used. The filled templates were dried and calcined at over 800 °C, which set the ceramic structure and removed the polymer material. In this way, non-porous ceramic monoliths were produced. Monolithic structures were the reverse replicas of the templates used for the production process. Some of the monoliths were additionally coated with one or more active catalytic components by impregnation [106,107]. Three-dimensional printing made it possible to build templates for monolithic catalysts with purpose-designed structures. The method allowed precise replication of the template structure and control over the monolith architecture on a scale of microns to prevent loss of geometric features and shrinkage [107].

Monoliths with a 3D system of channels were thus synthesized. The monoliths varied in the size of main channels. The catalysts contained Mn and/or Na_2_WO_4_ and were intended for oxidative coupling of methane (OCM). The Mn-Na_2_WO_4_ catalysts were active and selective in the tested process, providing high selectivity to C_2+_ hydrocarbons at 67–70% and conversion at 23–25%. These results were similar to those provided by the best Mn-Na_2_WO_4_/SiO_2_ powdered catalysts reported so far in the reference literature. The catalysts also revealed excellent stability during 20 h of the process. The size of the monolith channels significantly affected the catalytic performance, especially the distribution of the products of the process [107].

Monoliths with zeolite layers were also produced. The monolithic catalysts were dedicated for a process of gaseous phase α-pinene isomerization. The catalysts were synthesized on supports formed by corundum-silicate monoliths (without any doping) in the same way as above and functionalized by deposition of Mordenite Framework Inverted (MFI) zeolite layers with various atomic ratios of Si/Al. The activity of ZSM-5-coated monoliths increased with acidity (the number of acid sites), i.e., with reduction of the Si/Al ratio within the deposited layer. The highest α-pinene conversion was produced by the catalyst coated with a layer of ZSM-5 Si/Al = 30. The monoliths suffered partial deactivation over time, but it was possible to restore the initial activity by regeneration in an oxidative environment [108].

The monoliths prepared in the assistance of 3D printing with MFI zeolite layers were also used in the total oxidation of volatile organic compounds (VOCs) [109]. It was found that the deposition of cobalt by ion-exchange or impregnation techniques leads to the formation of highly dispersed Co_3_O_4_ spinel particles, resulting in excellent catalytic activity in the total oxidation of toluene. Moreover, the stability tests revealed that the obtained monolithic catalysts can work for a long time without noticeable changes in toluene conversion and selectivity to CO_2_.

Other research teams synthesized ceramic [110] and carbon [111] monoliths for catalytic applications by using methods similar to the ones discussed above. Microstereolithography was used to print the polymer templates. The templates were filled with cordierite paste, thermally processed, and sealed with Al_2_O_3_. The resulting monoliths had a honeycomb structure, with a different architecture of channels. In the last step of production, an active phase of CuO/CeO_2_ was loaded on these supports. The monolithic catalysts were tested by CO oxidation with excessive oxygen and preferential CO oxidation in an H_2_-rich mixture (CO-PROX). The catalyst with asymmetric channels achieved higher conversion in both reactions and improved the reaction rates [110].

Polymer templates were synthesized with a 3D ME printer. The printed templates were filled with a paste of phenol-formaldehyde resin, followed by solvothermal polymerization, and the residues of the templates was removed with a solvent. Next, calcination was run under a nitrogen atmosphere. The resulting carbon supports which varied in architecture had Ni-Al_2_O_3_ layers deposited. The obtained monolithic catalysts were tested in a process of CO methanation. To produce high catalytic performance, monoliths with straight channels with a diameter of approx. 0.67 mm were used. The monoliths with tortuous channels approx. 0.84 mm in diameter enhanced the catalytic activity. Compared with conventional powdered catalysts, the 3D-printed monolithic ones presented higher catalytic performance for the syngas to methane process [111].

## 4. Conclusions

Additive manufacturing technologies are promising processes because of their high efficiency, ease of application, and adaptability. The intensive development of new AM technologies, the decreasing proportion of price to resolution, as well as the elaboration of new building materials have led to many promising constructional, biomedical, consumer, and scientific applications.

In the field of catalysis, AM technologies were extensively explored to fabricate a variety of heterogeneous catalysts and supports. Three-dimensional printed structures can be optimized in terms of geometry and chemical composition as required by the target product to improve mass and heat transfer as well as their catalytic performance. This review describes some promising results connected with the architecture design of catalysts.

Currently, two approaches to the preparation of catalytic structures are being investigated. The first solution is direct production of materials, layer-by-layer, using inks containing catalytic materials or catalyst precursors. In the second indirect preparation method, a typical printed model made of polymer resin is used as a template (negative replica) to prepare a catalyst. Both approaches have advantages and disadvantages.

Further development is necessary in the following directions:

- improvement of the feedstock materials (reduction of temperatures and the number of post-processing operations),
- printing techniques (improvement of the resolution and the ability to create multilayer materials at once),
- control of porosity of catalytic materials prepared by AM,
- better control of acid-base and redox properties of catalysts,
- optimization of catalyst design.

## Figures and Tables

**Table 1:** Additive manufacturing classification by ASTM International [2,20,29,30,31].

Category	Operating Principle	Examples of Technology	Materials
Vat Photopolymerization (VP)	A liquid photopolymer is selectively cured in a vat by light-activated polymerization.	Stereolithography, Digital Light Processing, Continuous Liquid Interface Production	polymers, ceramics
Material Jetting (MJ)	Building material droplets are deposited selectively.	PolyJet, Multi-Jet, 3D Plotting	polymers, ceramics, composites, hybrid, biological
Binder Jetting (BJ)	A liquid binding agent is selectively deposited to bind the powdered material.	3D Printing	polymers, ceramics, composites, metals, hybrid
Material Extrusion (ME)	The material is selectively dispensed via a nozzle or an orifice.	Fused Deposition Modeling/ Fused Filament Fabrication/ Fused Layer Modelling, Robocasting/ Direct Ink Writing/ 3D Fiber Deposition	polymers, composites
Powder Bed Fusion (PBF)	Thermal energy is applied to selectively fuse areas of the powder bed.	Direct Metal Laser Sintering, Selective Laser Sintering/ Selective Laser Melting, Electron Beam Melting	polymers, ceramics, metals, composites, hybrid
Sheet Lamination (SL)	Sheets of the building material are bound with one another to form the object.	Laminated Object Manufacturing, Ultrasound Consolidation	polymers, ceramics, metals, paper, hybrid
Directed Energy Deposition (DED)	A focused flux of energy is applied to fuse materials by melting during deposition.	Laser Engineered Net Shaping, Direct Metal Deposition, Laser Powder Deposition, Electron Beam Additive Manufacturing	metals, hybrid
